# Phenolic Acids-Mediated Regulation of Molecular Targets in Ovarian Cancer: Current Understanding and Future Perspectives

**DOI:** 10.3390/ph16020274

**Published:** 2023-02-11

**Authors:** Nazia Nazam, Nasimudeen R. Jabir, Iftikhar Ahmad, Saif A. Alharthy, Mohd Shahnawaz Khan, Rashid Ayub, Shams Tabrez

**Affiliations:** 1 Amity Institute of Molecular Medicine & Stem Cell Research, Amity University, Noida 201301, Uttar Pradesh, India; 2 Department of Biochemistry, Centre for Research and Development, PRIST University, Vallam, Thanjavur 613403, Tamil Nadu, India; 3 Department of Biochemistry, Faculty of Science, King Abdulaziz University, Jeddah 21589, Saudi Arabia; 4 King Fahd Medical Research Center, King Abdulaziz University, Jeddah 21362, Saudi Arabia; 5 Department of Medical Laboratory Sciences, Faculty of Applied Medical Sciences, King Abdulaziz University, Jeddah 21589, Saudi Arabia; 6 Department of Biochemistry, College of Science, King Saud University, Riyadh 11451, Saudi Arabia; 7 Technology and Innovation Unit, Department of Science, King Saud University, Riyadh 11451, Saudi Arabia

**Keywords:** ellagic acid, gallic acid, ovarian cancer, phytochemicals, protocatechuic acid, salicylic acid

## Abstract

Cancer is a global health concern with a dynamic rise in occurrence and one of the leading causes of mortality worldwide. Among different types of cancer, ovarian cancer (OC) is the seventh most diagnosed malignant tumor, while among the gynecological malignancies, it ranks third after cervical and uterine cancer and sadly bears the highest mortality and worst prognosis. First-line treatments have included a variety of cytotoxic and synthetic chemotherapeutic medicines, but they have not been particularly effective in extending OC patients’ lives and are associated with side effects, recurrence risk, and drug resistance. Hence, a shift from synthetic to phytochemical-based agents is gaining popularity, and researchers are looking into alternative, cost-effective, and safer chemotherapeutic strategies. Lately, studies on the effectiveness of phenolic acids in ovarian cancer have sparked the scientific community’s interest because of their high bioavailability, safety profile, lesser side effects, and cost-effectiveness. Yet this is a road less explored and critically analyzed and lacks the credibility of the novel findings. Phenolic acids are a significant class of phytochemicals usually considered in the nonflavonoid category. The current review focused on the anticancer potential of phenolic acids with a special emphasis on chemoprevention and treatment of OC. We tried to summarize results from experimental, epidemiological, and clinical studies unraveling the benefits of various phenolic acids (hydroxybenzoic acid and hydroxycinnamic acid) in chemoprevention and as anticancer agents of clinical significance.

## 1. Introduction

Cancer is a global concern and one of the significant hurdles toward achieving desirable life expectancy worldwide [1,2]. With the dynamic rise in the number of cancer cases, it is one of the leading causes of death worldwide, accounting for 10 million deaths in 2020 [3]. Among innumerable cancers, ovarian cancer (OC) is the seventh most diagnosed malignant tumor, while among the gynecological malignancies, it ranks third after cervical and uterine cancer and sadly bears the highest mortality and worst prognosis [4,5]. Though this malignant neoplasm has a lower prevalence than breast cancer, it is three times more fatal compared to the former. It is striking to note that the number of diagnosed cases and the mortality rate is predicted to increase by almost 42–50% by 2040, resulting in enormous global burden [3].

Of all the malignant ovarian tumors, greater than 90% have an epithelial origin, thus making epithelial ovarian cancer (EOC) the predominant histotype [6]. The five major EOC pathologic subtypes (high-grade serous, endometrioid, clear cell, mucinous, and low-grade OC) differ in their origin, clinic pathology, molecular level, gene expression profile, and prognosis [7]. The other subtypes based on the origin include sex-cord stromal, germ-cell, and mixed-cell types [6]. Though females of all ages could develop ovarian carcinogenesis, it is most commonly diagnosed in postmenopause women. This is an alarming situation since more than 75% of affected women are diagnosed at an advanced stage. Late diagnosis is attributable to the asymptomatic early stage, while the symptoms of the late-stage disease are nonspecific [8]. Despite continuous innovations and advancements in surgical and systemic treatments, the 5-year overall global survival rate of OC remains as low as 11% for stage IV disease compared with 88% for stage I disease [9]. This is because the disease typically appears at a very late stage, and unfortunately, there is not much improvement in these statistics [10].

Ovarian cancers at an advanced stage are too aggressive, and available therapeutics are inefficient due to acquired chemoresistance upon progression, recurrence risk, and in many cases production of severe toxicological effects [11]. The treatment thus remains challenging irrespective of the total positive response with surgical resection and platinum/taxanes-based chemotherapy by the intravenous pathway, with diminutive effectiveness in advanced stages [12,13].

Multiple cytotoxic and synthetic drugs have been used as first-line treatments, yet they have failed to show significant survival benefits. They are associated with side effects, risk of recurrence, and drug resistance. Novel strategies to combat ovarian cancer are urgently needed for improved survival, and the research to unravel new routes of administration and potent therapeutic candidates for this malignancy is essential. In search of novel therapeutics, there is a paradigm shift from synthetic to plant-based anticancer agents, which gained attention lately.

**Figure 1:**
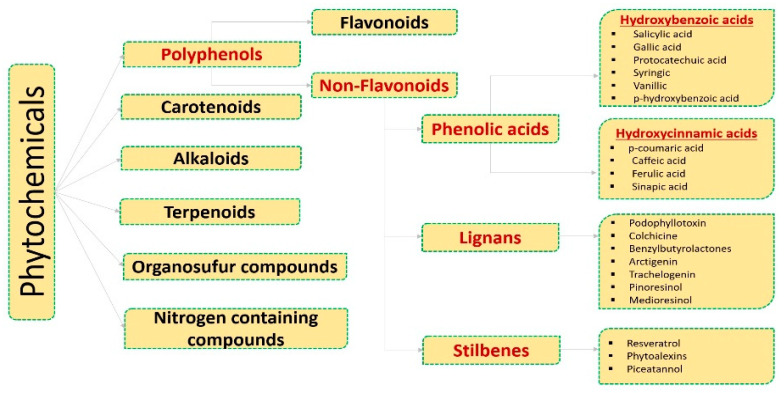
Classification of natural phytochemicals: Broadly classified into six major classes. Polyphenols is one major class further subdivided into two—flavonoids and nonflavonoids. Phenolic acids, lignans, and stilbenes are three distinct subclasses of nonflavonoids. Hydroxybenzoic and hydroxycinnamic acids further comes under the subclass of phenolics acids, while the other two subclasses include stilbenes and lignans. (Classification adopted and redrawn from [14,32,33]).

Phytochemical is a broad term referring to plant (phyto) chemicals. These are a wide variety of bioactive non-nutrient compounds that occur naturally in fruits, vegetables, grains, and other plant foods with desirable health benefits beyond basic nutrition and that could help prevent chronic diseases, including cancer. The last 3–4 decades have witnessed a plethora of scientific studies focused on phytochemicals, such as flavonoids, nonflavonoids, carotenoids, phenols, nitrogen-containing, and organosulfur compounds (Figure 1), with diverse therapeutic potential against various cancer types [14,15,16,17,18,19,20]. Numerous epidemiological studies have also observed reduced cancer incidence with the use of phytochemicals [14,21]. In a population-based cohort study in female breast cancer patients, fruit and vegetable consumption have been linked with lower cancer risk [22]. The important role of a plant-based diet in reducing risks of hormone-related neoplasms, such as ovarian cancer, is also well established [23].

The major category of phytochemicals includes phenolic compounds comprising flavonoids and nonflavonoid subtypes. Phenolic acids comprise an abundant subgroup of the phenolic compounds bearing the basic chemical structure of C6-C1 as hydroxybenzoic or C6-C3 as hydroxycinnamic acids, along with a phenolic ring and a carboxyl substituent. The biosynthesis of phenolic acids is regulated by the shikimic acid or phenylpropanoid pathway of plant metabolism. The anticancer effect of flavonoids has been vastly studied for a long time, and updated reviews underscoring the role of various flavonoids have been published to date [24,25,26,27]. The role of such dietary flavonoids against ovarian cancer and reduced risk has also been significantly studied [23,28,29]. A prospective study suggested a reduced EOC risk upon intake of five common dietary flavonoids, viz., myricetin, kaempferol, quercetin, luteolin, and apigenin [30]. A meta-analysis suggested that dietary flavonoids and subtypes (isoflavones, flavonols) confer protection against ovarian cancer with a reduced risk of recurrence [29]. Studies continue to highlight the anticancer property of flavonoids, such as quercetin, kaempferol, myricetin, galangin, and isoliquiritigenin, that include modulating intrinsic apoptotic pathway, MEK/ERK and STAT3 pathways; downregulating MDR-1; apoptotic induction via p53-dependent pathways; and suppression of the epithelial-to-mesenchymal transition (EMT) [14,23,28].

The vast literature on flavonoids indicates their potential in OC chemoprevention and treatment. Studies on the effectiveness of phenolic compounds (nonflavonoids subtypes) in ovarian cancer have also sparked interest in recent times. Studies are emerging because of their high bioavailability, safety profile, and lesser side effects [31]. Yet this is a road less explored and critically analyzed and needs more credibility of the novel findings. Hence, we tried to summarize results from experimental, epidemiological, and clinical studies unravelling the benefits of plant-based phenolics (nonflavonoids) in chemoprevention and as anticancer agents of clinical significance. Among the various nonflavonoids, we have limited ourselves to cover the anticancer efficacy of different phenolic acids only in this article to keep readers’ interest and not to make this article easy to grasp. In this review, we have also briefed the targets of these phenolics in ovarian carcinogenesis and the mechanisms involved in regulating the function of these targets, which could serve useful in future drug development.

## 2. Phenolic Acids Involved in the Regulation of Molecular Targets

### 2.1. Hydroxybenzoic Acids

#### 2.1.1. Gallic Acid

Gallic acid (GA) is the most popular hydroxybenzoic acid member among the phenolic acids. It is a secondary polyphenolic metabolite of plant, also known as 3,4,5-trihydroxybenzoic acid. GA is particularly abundant in processed beverages, such as red wine and green tea, while in its free state, as phenolic acid polymers (condensed tannins), it is present in the berry/tea leaves, pomegranate root bark, gallnuts, oak bark, and many other fruits and vegetables. GA and its ester derivatives are widely used for an edible purposes, as flavoring agents, and as preservatives in the food industry [34]. Besides their edible use, there are diverse scientific reports on their biological and pharmacological activities, such as antioxidant, antibacterial, antiviral, anti-inflammatory, anticancer, and protective effects against cardiovascular, neuropsychological, gastrointestinal, and metabolic diseases [35,36,37]. The antitumor activity of GA is reported in various female cancers, such as its potentiating chemotherapeutic effect of paclitaxel in cervical cancer [38], while it is observed to potentiate paclitaxel and carboplatin effect in breast cancer [39]. Anticancer studies highlight its medicinal importance, validate the use of these traditional herbs in tumor prevention and therapy, and show that gallic acid may potentially serve as a candidate for female cancer treatment [40,41].

Preliminary studies on the cytotoxic effect of GA against ovarian cancer cells (OVCAR-8, A2780 and its cisplatin-resistant form—A2780cis) offer it as a potent anticancer agent [42]. In one study, eight natural phenols from traditional Chinese medicine, including -(−)-epicatechin, (+)-catechin hydrate, ellagic acid, tangeretin, nobiletin, baicalin, baicalein, and GA were screened, and GA was one of the potent agent to inhibit proliferation of human ovarian cancer cell lines (OVCAR-3 and A2780/CP70—a cisplatin-resistant) [43]. Gallic acid selectively inhibited these ovarian cancer cells but did not affect normal ovarian cells—IOSE 364 [44]. The mechanism of action of GA on ovarian cancer angiogenesis was found to be the inhibition of vascular endothelial growth factor (VEGF) secretion. Moreover, the elevation in PTEN expression (a tumor suppressor gene) and downregulation of AKT phosphorylation and HIF-1α expression suggests the role of PTEN/AKT/HIF-1α pathway. These findings indicate the high potential of gallic acid in the prevention of ovarian cancer since the therapeutic activity of GA is well tolerated by the normal ovarian cell lineage.

A recent study observed a significant decrease in cell viability in SKOV-3 and OVCAR-3 (50 and 94 μg/mL, respectively) with GA, along with morphological changes mainly in the actin/tubulin cytoskeleton, cell cycle arrest, cell death by apoptotic induction, and ROS generation [45]. In silico analysis employing a similarity ensemble approach model found possible GA inhibition of the carbonic anhydrase IX protein—a zinc-dependent metalloenzyme regulating intracellular pH [46]. The in vivo validation of this study with xenograft mice model revealed inhibitory effects up to 50% on tumor lesions development with GA administered by peritumoral (p.t.) route along with decreased vascularity, necrotic/fibrotic areas, neoplastic stroma retraction, and apoptosis. It is interesting to note that these results resemble those attained by the group that received paclitaxel. The p.t. administration of GA did not show behavioral changes or any toxicity signs. Thus, therapeutic targets of GA could possibly be attributed to its potential to induce ATM/Chk2/p53 activation, COX-2/NF-kB, and GSH inhibition [47]. Developing new medications to block these target proteins in ovarian cancer is an intriguing perspective that should be explored. However, histological and paraclinical analysis of mice organs and blood revealed GA-induced hepatic necrosis, leukocyte infiltration, hypertransaminasemia, and hypoazotemia, all of which are linked to hepatic failure brought by chronic inflammation caused by the destruction of liver parenchyma, highlighting the requirement of additional studies to find an adequate therapeutic dose for GA. Further study was conducted on the proliferation suppressive role of GA on platinum-resistant OVCAR-3 and A2780/CP70 OC cell lines. This was attributed to GA-induced caspase-3 mediated apoptosis by upregulating the proapoptotic proteins (Bax and Bad) through p53 protein. Additionally, it also induced cell cycle arrest via the p53-p21-Cdc2-cyclin B pathway in these cells [48]. Considering that GA has good bioavailability, it can potentially be employed toward the treatment of OC; however, further verification of the in vivo model is required. The cytostatic action of paclitaxel (PTX) is increased in two OC cell models, namely the A2780 doxorubicin-sensitive cells and the drug-resistant variant A2780AD upon cotreatment with GA. In addition, the growth inhibition potential of PTX was reduced in A2780AD cells, concordant with the multidrug resistance phenotype. GA potentiated the PTX-induced G2/M phase arrest and acted as a chemo-sensitizer. Interestingly, GA-sensitized PTX-resistant OC cells resulted in ROS-mediated inactivation of ERK; hence, it could be effective as a co-adjuvant to PTX in ovarian carcinoma treatment [49]. The combined therapy of PTX and GA might signify a novel strategy against cancer resistance, necessitating lower doses contrasted to paclitaxel alone. Isolated polysaccharides from *Ganoderma lucidum* with polyphenolic content as GA equivalents significantly reduced malondialdehyde production; reduced oxidative stress such as SOD, CAT, GSH-Px, TAOC activity; and increased serum antioxidant enzymes activity in serum of ovarian cancer rat model. These results suggest that the antioxidant activity might benefit ovarian cancer therapy [50].

#### 2.1.2. Salicylic Acid

Salicylates usage dates back to ancient times for its varied medical benefits, the simplest one being attributed to salicylic acid’s (SA) diverse therapeutic application [51]. The existence of salicylates in innumerable plant products of dietary benefits spanning a wide range of foods and beverages, such as berries (blue, boysen, logan), apricots, dates, raisins, oranges, cucumbers, and black tea, with the richest being condiments (aniseed, cumin, hot paprika, and thyme) [52]. Salicylic acid is a prototype drug and of late is replaced by its synthetic derivatives—aspirin and salsalate, which are converted to salicylate upon metabolism [53]. The extensive medicinal use of aspirin ranges from its analgesic effect to controlling blood pressure. The underlying mechanisms are its interference with various proinflammatory modulator genes and activation of AMP and AMPK 11, while inhibiting kinase activity [54]. Furthermore, salicylates at large regulate cellular oxidative stress by ROS generation and work as free radical scavengers [55]. Some of these effects have been correlated to a putative anticancer effect of aspirin and its metabolic product in humans [56,57]. Several studies on salicylic acid and its derivatives (acetylsalicylic and aspirin) have been carried out that delineate the potential molecular mechanisms behind their chemopreventive properties in many cancers [56,57,58]. Given the proven safety record and modulating effect on signaling pathways, the possibility of using salicylic acid and its derivative for ovarian cancer treatment could be considered.

A salicylic acid derivative niclosamide effectively inhibits ovarian cancer cell (SKOV3, HeyA8) proliferation, migration, cell cycle progression, and induced apoptosis [59]. The multiple signaling pathways for its anticancer role include modulation in ELK1/SRF, AP-1, MYC/MAX, and NF-кB. However, silencing of IGF signaling sensitized niclosamide-induced antiproliferative and anticancer activities both in vitro and in vivo. Hence, this drug could be used as a repurposed anticancer agent and holds potential as a combination therapy for treating human cancers, including ovarian cancer.

Several pieces of scientific evidence confirm niclosamide as an anticancer agent capable of inhibiting various cellular pathways identified by high-throughput screening platforms. Yet its direct binding interactions with discrete biological molecule(s) are not established. Quite recently, 2 RNA-binding proteins (RBPs)—FXR1 and IGF2BP2—were identified as key signal transduction mechanisms altered by niclosamide in ovarian cancer [60]. Using ovarian cystadenocarcinoma TCGA data sets (591 patients), significant correlation was observed with high expression of RBPS associated with reduced survival of ovarian cancer patients. In contrast, silencing these proteins significantly reduced the percentage of viable cells and modulated cell adhesion and migration. FXR1 and IGF2BP2 were thus suggested as direct targets of niclosamide with a critical role in driving multiple oncogenic pathways in ovarian carcinoma. Using stem-like ovarian tumor-initiating cells (OTIC), niclosamide was identified as one of the hits that specifically targeted OTICs. It may do this by interfering with several metabolic processes, including biogenesis, biogenetics, and redox regulation [61]. This study supports niclosamide as a promising therapy for ovarian cancer and warrants further preclinical and clinical evaluation of its safety. In most of the tumor cells isolated from the 34 patients’ ascites with primary ovarian cancer, niclosamide and carboplatin exhibited greater cytotoxicity than either alone, and their mechanism of action involved inhibiting the Wnt/β-catenin pathway [62]. Another study indicated that this drug may also exert its anticancer activity through inhibition of Wnt/β-catenin and its direct downstream target gene FGF1 influences its sensitivity in ovarian cancer [63].

The niclosamide-like analog showed cytotoxicity alone and/or in combination with carboplatin against tumor spheres from ovarian cancer patient ascites and slices from solid tumor samples. The downregulation of Wnt pathway-associated proteins in patient samples treated with niclosamide analogs was observed [64]. These results suggest that more soluble niclosamide analogs may be useful for the treatment of ovarian cancer in combination with existing chemotherapy. Niclosamide-targeted ovarian cancer-stem cells (CSCs) inhibit several altered signaling pathways associated with cancer metastasis and recurrence [65]. It preferentially targets crucial pathways, such as Wnt, mTOR, and STAT3, as well as reverse platinum resistance. A recent study highlighted another novel anticancer mechanism of this drug involving cell metabolism interruption [66]. Treating human ovarian cancer cells (SKOV3, HO8910) with niclosamide significantly inhibits cell growth and induces cellular apoptosis by inactivating MEK1/2-ERK1/2-facilitated signaling. Additionally, its treatment reduced mitochondrial respiration and aerobic glycolysis while dramatically enhancing ROS-activated and JNK-mediated apoptosis in cells deprived of glucose. Niclosamide also showed an anticancer effect in the nude mouse transplanted tumor model.

Niclosamide is a potent mitochondrial uncoupler that can affect cell cycle arrest and apoptosis [67]. One criticism for using niclosamide as an anticancer drug is its poor water solubility and bioavailability [65]. As a result, a novel nanosuspension of niclosamide (nano-NI) was evaluated in ovarian cancer and was found to efficiently inhibit the growth of these cells [68]. This nanoformulation induced an altered metabolic phenotype shift from oxidative phosphorylation to glycolysis in vitro (CP70 and SKOV3) and also repressed tumor growth short of noticeable toxicity in mice. A postoral administration pharmacokinetic study showed its rapid absorption (attaining the maximum plasma concentration in 5 min) and improved the bioavailability in rats. Hence, nano-NI holds potential as a new ovarian cancer treatment modality, but further clinical studies are warranted.

#### 2.1.3. Ellagic Acid

Ellagic acid (EA) is another polyphenolic acid extensively studied for its beneficial effect against different types of cancer. It is a dimeric gallic acid derivative found either in freeform or as a complex within the ellagitannins family. EA is widely present in fruit (pomegranate, grape), berries (blackberry, raspberry strawberry, cranberry, and blueberry), nuts (walnuts, chestnuts, almonds), and dry fruits [69]. Its anticancer properties are mainly accredited to its inhibition in proliferation, cell migration, invasion, angiogenesis, and induction of apoptosis [70]. EA also enhances cancer cell sensitivity to therapy (chemotherapy/radiotherapy) by reducing the toxic effects of ROS-generating chemotherapies due to its antioxidant properties [71]. Small clinical trials on colorectal cancer (CRC) patients have suggested EA as supportive therapy to standard chemotherapy, and it modulates the expression of genes and microRNAs in CRC [72,73].

Yuan-Chiang and co-workers studied EA’s antimalignancy and antichemoresistance properties in human OC [74]. When OC cells—ES-2 and PA-1 cells—were treated with EA (10~100 μM), it was observed that it inhibited cellular proliferation, induced G1-arrest due to elevated p53 and Cip1/p21, and decreased levels of cyclin D1 and E. Additionally, it induced apoptosis mediated by caspase-3 via an increased Bax: Bcl-2 ratio and also restored anoikis in these cell lines. EA-mediated apoptotic induction and autophagy inhibition enhanced the efficacy of EA chemotherapy.

EA significantly reduced A2780 OC cell proliferation, migration, and invasion in vitro, and its treatment inhibited tumor growth without noticeable side effects in vivo in nude mice. EA could inhibit the metastasis by downregulating MMP-2 and MMP-9 expression since matrix metalloproteinases (MMPs) are endopeptidases, capable of extracellular matrix (ECM) degradation, hence promoting migration, invasion, and metastasis of cancer cells [75]. Engelke et al. [76] demonstrated that permanent treatment of nontoxic EA concentration prevented cisplatin resistance in A2780 OC cell lines. On the contrary, its absence resulted in resistance to cisplatin of clinical relevance in these cells. However, in the resistant isoform A2780CisR, EA could not reverse cisplatin resistance, albeit cell proliferation and migration were observed to be reduced. Reduced ErbB2 and ErbB3 phosphorylation in EA-treated cells might be accountable for decreased OC cell migration and chemoresistance prevention or for retaining a sensitive phenotype.

EA also caused apoptotic cell death of doxorubicin-resistant NCI/ADR-RES ovarian cancer cells, and it was elucidated that JNK and Akt phosphorylation are indispensable in EA-induced apoptosis [77]. In a preliminary study employing OVCAR-3 and A2780/CP70 OC cells, EA was identified as a compound capable of proliferation suppression and moderate inhibition of VEGF secretion, a growth factor that induces blood vessel growth [43]. The antitumorigenic potential of ellagic acid confirmed in SKOV-3 OC cells involved cytotoxic autophagy activation mediated by Akt inhibition and AMPK activation. Mechanistically, it decreased the levels of the two downstream targets, mTORC1 and p-Akt, but increased the levels of AMPK (Thr172) [78]. PI3K/Akt inhibits autophagy by activating mTORC1, and AMPK inhibits mTORC1, thus activating autophagy. Thus, the key role of dysregulated autophagy in ovarian cancer development and progression indicates its potential as a promising therapeutic target. The nanoformulations of EA with enhanced solubility and bioavailability could also be developed into EA-based therapeutics.

#### 2.1.4. Protocatechuic Acid

Protocatechuic acid (PCA), 3,4-dihydroxybenzoic acid is a benzoic acid-derived phenolic acid, widely distributed in edible plants, fruits, nuts, green tea, and black rice [79]. PCA is a highly prevalent, biologically active component in the human diet, present in *Oryza sativa* and *Allium cepa* [80]. PCA is also found in plums (*Prunus domestica*), gooseberries (*Ribes uva-crispa*), grapes (*Vitis vinifera*), almonds (*Prunus amygdalus*), olive oil, honey, soybean, star anise (*Illicium verum*), melissa (*Melissa officinalis*), medical rosemary (*Rosmarinus officinalis*), and cinnamon (*Cinnamomum aromaticum*) [80,81]. It is a bioactive constituent of medicinal plants, such as *Hibiscus sabdariffa*, *Ginkgo biloba*, *Hypericum perforatum*, *Cibotium barometz*, *Stenoloma chusanum*, *Ching,* and *Ilex chinensis Sims* [80,82]. It is incompatible with potent oxidizing agents and has limited water solubility (1:50) but has fair solubility with alcohol and ether [82].

PCA has been reported to trigger caspase-mediated apoptosis, possess antioxidant activity, and augment the cytotoxicity against multiple cancer cell lines [79]. A growing body of evidence demonstrated that PCA has an influential antioxidative effect by reducing lipid peroxidation and increasing the scavenging of hydrogen peroxide and diphenylpicrylhydrazyl. Furthermore, glutathione peroxidase and superoxide dismutase activity is increased by PCA, whereas xanthine oxidase and NADPH oxidase activity are reduced, resulting in lower malondialdehyde concentrations [81]. PCA is thought to be the best peroxyl radical scavenger in the polar environment of aqueous solutions. In contrast, in the nonpolar environment of lipid solutions, it is believed to be a reasonable antiradical protector [83]. PCA has also been reported for other anticancer-associated mechanisms, such as induction of cytotoxicity, suppression of Bcl-2 gene expression, retinoblastoma phosphorylation, and c-Jun N-terminal kinase-dependent signal transduction in various human cancer cells [81]. Black raspberries’ anthocyanin and PCA have been shown to reduce esophageal carcinogenesis brought on by N-nitroso-methyl benzylamine by inhibiting COX-2, iNOS, p-NF-kB, and she biomarkers and modulate PTX3 cytokine expression [84]. DBP-diol-induced DNA adduct formation and mutagenesis were prevented by pretreatment with PCA via enzyme inhibition or phase 2 enzyme stimulation [85]. By inducing ROS reduction, p53-independent apoptosis, and autophagy, PCA reduces the growth of ovarian cancer cells [86]. Evidence from in vitro studies reported cell-protective effects of PCA through various mechanisms such as increased IkB degradation and consequent NF-kB activation in TNF-*α*-induced cell death of various cancer tissues [87]. Attenuated changes in the mitochondrial membrane permeability, decreased oxidative stress damage, increased Bcl-2 levels, decreased caspase-3 activity, and reduced LDH leakage have also been reported in various cellular models treated with PCA [86,87]. The PCA treatment in different experimental models showed inhibition of cellular proliferation and induction of apoptosis via reduced expression of Bcl-2, increased Bax level, activated JNK/p38 MAPK, and Fas/FasL pathways, and an inhibitory effect on genes associated with inflammation [84,87,88].

Oxidative stress tolerance is a significant factor in ovarian carcinogenesis at several stages of the disease, including its onset, development, invasion, and metastasis [89]. Moreover, oxidative stress control has been considered one of the noteworthy therapeutic targets in OC. The antioxidant effect by PCA indicates its therapeutic potential against OC. Multiple clinical trials have been registered to evaluate the effectiveness of antioxidants in treating OC [90,91]. The PCA influencing targets include Bcl-2, c-Jun *N*-terminal kinase, COX-2, iNOS, p-NF-kB, IkB, TNF-*α*, MPP, caspase-3, LDH, p38, MAPK, and PTX3, which are the well-established anticancer drug targets [31,79]. Roselle extract with PCA was shown to have an effective antiproliferative effect on Caov-3 ovarian cells [92]. Gouveia et al. [93] reported the beneficial effects of PCA after cisplatin-induced ovarian toxicity in mice. PCA at 20 mg/kg induced apoptosis, maintained cell proliferation and mitochondrial function, reduced ROS production, and increased GSH expression through PTEN and FOXO3a proteins [93]. Earlier research by Xie et al. [86] revealed that PCA treatment dramatically decreased the viability of cells and their capacity to form colonies in OVCAR3, SKOV3, and A2780 cells. PCA treatment caused G2/M phase cell cycle arrest, activated PARP and caspase 3, and upregulated and downregulated Bax and Bcl2, respectively, in OVCAR3 cells [31]. PCA treatment dramatically increased the autophagy-related protein LC3-II and promoted the production of GFP-LC3 puncta. Additionally, PCA and an autophagy inhibitor co-treatment reduced the cytotoxicity that PCA induced in OVCAR-3 cells. PCA may inhibit OVCAR3 cells since it enhances glutathione levels intracellularly and lowers intracellular ROS. Overall, PCA might be used as a chemopreventive agent for ovarian cancer since it may affect apoptosis and autophagy.

#### 2.1.5. Syringic Acid

Syringic acid (4-hydroxy-3,5-dimethoxybenzoic acid, SyA) is 3,5-dimethyl ether derivative of gallic acid and a conjugate acid of a syringate. It belongs to the family of hydroxybenzoic acids, which has been demonstrated to promote condensation, polymerization, and oxidation processes. SyA has been found in various dietary foods, including dates, olives, pumpkin, grapes, spices, acai, red wine, palm, and honey [31]. Several studies reported favorable biological properties of SyA, such as, minimal toxicity, wide diffusion in nature, and intriguing pharmacological effects [94,95,96]. It displays a broad range of therapeutic promise in mitigating various clinical conditions, viz., diabetes, cardiovascular disorders, cancer, and cerebral ischemia [94]. Due to the structural advantage of two methoxy moieties linked to the aromatic ring at three and five positions, SyA shows an enhanced free radical scavenging capacity compared to many other phenolic acids [31]. Srivastava et al. [97] reported the free radical scavenging capacity of SyA with 2, 2-diphenyl-1-picrylhydrazyl (DPPH) and β-carotene. In another study, Cikman et al. [98] observed that SyA alleviates the oxidative stress markers and prevents L-arginine-induced rat acute pancreatitis. In one study, Ha et al. [99] reported the potential chemopreventive action of SyA on human epidermal keratinocytes (HaCaT) cells via the Nox/PTP-κ/EGFR axis. The SyA was found to suppress multiple UVB-induced events, including cyclooxygenase-2, matrix metalloproteinase-1, prostaglandin E2 expression, activator protein-1 activity, phosphorylation of MAPK and Akt, EGFR, and ROS formation [99]. In another study, Kowalczyk et al. [100] reported the anticancer potential of root extracts of *Menyanthes trifoliate* containing SyA with an induction of apoptosis mediated by G2/M phase cell cycle arrest and changes in the expression of Bax, Bcl-2, cas-3, and p53 as well as a reduction in the mitochondrial membrane potential in IV glioma cells. Afrin et al. [101] reported potential anticancer effects of manuka honey (a good source of SyA), including cell cycle arrest and apoptosis induction, suppression of *p*-Akt, and increased expression of p-p38MAPK. Likewise, another study reported that isolated SyA raised the expression of various molecules associated with the mitochondrial pathway, including cytochrome c, p53, Bax, apoptotic protease activating factor-1, caspase 3, and caspase 9, and downregulated Bcl-2 and generation of ROS in HepG2 cells [102]. SyA was evocative to be used as a prospective therapeutic drug with potential application for the treatment of OC due to the encouraging anticancer effect. Yang et al. [103] reported SyA-mediated suppression of STAT3/JNK/AKT pathway along with significant growth inhibition in human ovarian teratoma cancer cells (PA-1). SyA significantly modified the apoptosis-related protein expression level, viz., caspase-3, 8, 9, Bax, and Bcl-2 in PA-1 cells. Furthermore, SyA treatment significantly reduced the levels of cancer promoting proinflammatory cytokines TNF, IL-2, IL-6, and IL-10 in ovarian cancer cells [103]. The chemoprotective action of SyA on cyclophosphamide-induced ovarian damage via an inflammatory pathway was also reported by Liu et al. [104]. According to this study, SyA significantly decreased different anticancer parameters such as nitric oxide, myeloperoxidase, catalase, glutathione, glutathione peroxidase, superoxide dismutase, and malondialdehyde levels in both serum and ovarian tissue. In addition, SyA suppressed ovarian cancer parameters, including luteinizing hormones, antimullerian hormone, estradiol, and follicle-stimulating hormone and ovarian follicles. Moreover, SyA significantly downregulated cytokines, inflammatory mediators, and caspase-3 parameters [104]. Table 1 summarizes the major plant sources of these phenolic acids.

#### 2.1.6. Vanillic Acid

**Figure 2:**
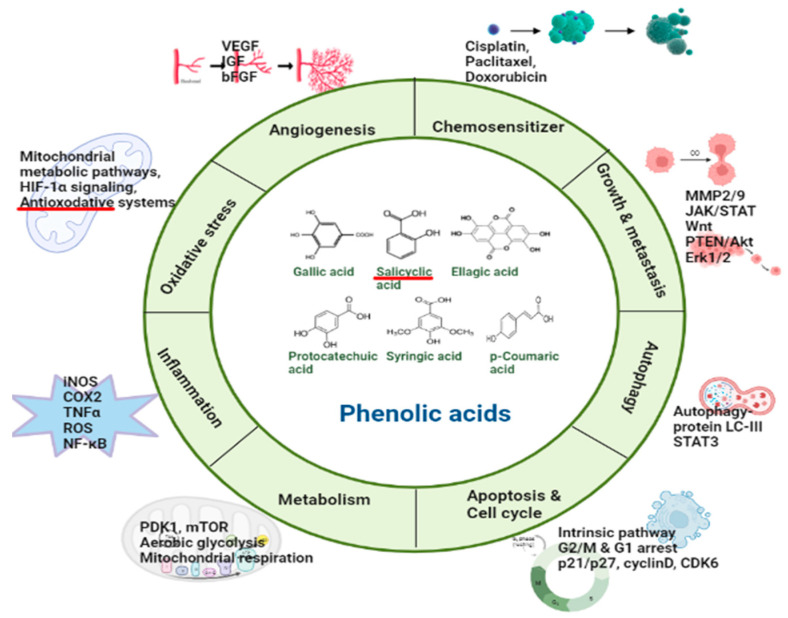
Signaling pathways associated with various biological process and the molecular action of phenolic acids on the ovarian cancer cell signaling pathways. (Created using Biorender software).

Vanillic acid (4-hydroxy-3-methoxybenzoic acid, VA), a conjugate acid of vanillate, is a major active compound isolated from *Angelica sinensis* and green tea [105]. VA is an oxidized form of vanillin and has been known as a common flavoring agent in food industries due to its pleasant odor [110]. Various studies observed a wide range of biological effects associated with VA against clinical conditions that includes cancer, diabetes, obesity, and neurodegenerative and cardiovascular disorders [111,112]. Owing to these benefits, VA has great promise to use as a therapeutic agent. However, the limited bioavailability due to its rapid elimination from the plasma is a significant concern [105]. Although there is limited research on the therapeutic effects of VA on OC, studies have shown that VA interacts significantly with several targets to have therapeutic benefit against OC [113,114,115]. Gong et al. [113] reported a VA-associated G1 phase arrest and inhibited the proliferation of human colon cancer in vitro and in vivo through different mechanisms, including suppression of HIF-1α and inhibition of mTOR/p70S6K/eIF4E binding protein 1 as well as Raf/MEK (mitogen-activated protein kinase)/ERK pathways. VA was able to induce cytotoxic and antiproliferative activity on NCI–H460 lung cancer cells via cell cycle arrest, mitochondria-mediated apoptosis, and DNA damage [116]. Bhavani and Subramanian reported a chemopreventive efficacy of VA against endometrial carcinoma in female rats attributed to reduced lipid peroxides and cytochrome P450 and an induced reversal of matrix metalloproteinases [114]. Likewise, Sitarek et al. [115] reported a VA-mediated inhibition of cell cycle progression via cell cycle arrest and stimulated p53-induced apoptosis in human glioma cells. It was also reported to induce enhanced mRNA levels of caspases 3, 8, and 9 and reduced the mRNA levels of Bcl-2 in glioma cells. The Bcl-2 family has been reported to play a crucial role in the chemoresistance of ovarian cancer, and the molecules target Bcl-2 have shown efficacy in overcoming the chemoresistance of ovarian cancer [117]. In addition, it has been demonstrated that VA inhibits multiple signaling pathways, preventing angiogenesis and suppressing cellular proliferation such hosphorpho-p70S6hosphorpho-mTOhosphorpho-4E-BP1, p-eIF4hosphorpho-c-Rahosphorpho-MEK1/2, ahosphorpho-ERK1/2 [105,118]. Figure 2 summarizes various biological processes and the molecular action of phenolic acids on the ovarian cancer cell signaling pathways.

### 2.2. Hydroxy Cinnamic Acids

#### 2.2.1. Caffeic Acid

Caffeic acid (CA) is a phenolic acid derived from hydroxycinnamic acid commonly found in an everyday dietary ingredient of humans [119,120]. It is a polyphenol and a secondary metabolite obtained from natural sources such as olives, berries, potatoes, and carrots but especially from coffee beans [106]. CA is found in various forms, such as organic acid esters, sugar esters, amides, and glycosides, or in more complex forms, such as dimers and trimers. It may also be bound to proteins and other polymers [106]. CA undergoes widespread metabolic pathways in the liver and kidney and showed an excellent safety profile in a phase 1 clinical trial [121,122]. The majority of in vitro and in vivo studies reported favorable physiological effects of CA and its derivatives, including antibacterial, antiviral, antioxidant, anti-inflammatory, antiatherosclerotic, antidiabetic, and anticancer, activities [106,122,123,124,125,126]. The use of CA helps decrease the chemotherapy side effects, increase treatment effectiveness, and decrease drug resistance [127,128].

The anticancer action of CA and its derivatives are attributed to different mechanisms of action, such as prevention of ROS formation, diminishing the angiogenesis of cancer cells, enhancing the tumor cell DNA oxidation, blocking STATS (transcription factor and signal translation 3), and suppressing MMP-2 and MMP-9 (collagen IV metalloproteases). The antioxidant and prooxidant property of CA is functionally attributed to its chemical structure as the presence of free phenolic hydroxyls, and the double bond in the carbon chain enables the elimination of free radicals and prevention of ROS production [106,125]. The free phenolic ortho-dihydroxyl group in CA has been shown to reduce the enthalpy of hydroxyl bond dissociation and increase the transfer rate of H-atoms for peroxyl radicals along with the number and location of phenyl rings. Likewise, the 2,3 double bond in the unsaturated carbon side chain increases the stability of the phenolic radical [122,125,129]. It is interesting to note that the iron-chelating potential of CA has also been linked to its antioxidant activity, with iron-CA complexes reducing Fenton-induced oxidative damage by blocking the generation of free hydroxyl radicals [123]. It is well-known that nitro compounds, such as nitrosamines and nitrosamides, are the significant inducers of cancer pathology [124]. Dietary components that include CA have been shown to protect against carcinogenesis by reducing the formation of nitro compounds. MMP-2 and MMP-9 are two important matrix metalloproteases expressed in tumor cells, which promote carcinogenesis by destroying the extracellular matrix type IV collagen at the basement membrane during cancer invasion and metastasis [106]. CA has also been reported as influencing the regulation of the mRNA levels of iNOS, COX-2, TNF-α, NF-κB, NFAT, and AP-1 [130]. The oncogene targeting by CA has been reported in various cancer types [131,132]. CA exhibited a vital role in preventing tumor progression via cell cycle modulation, inhibition of colony formation, and changes in the expression of caspases [31,133]. CA has also been reported to inhibit TGFβ-SMAD2 signaling pathway mediated by microRNA-148a in in vitro and in vivo models [31,134].

Sirota et al. [135] reported a VA-associated G1 phase arrest and inhibited the proliferation of human colon cancer in vitro and in vivo through different mechanisms, including suppression of HIF-1α and inhibition of mTOR/p70S6K/eIF4E binding protein 1 as well as Raf/MEK (mitogen-activated protein kinase)/ERK pathways. However, a preincubation with CA before cisplatin treatment led to acquired resistance to cisplatin and reduced DNA binding. Moreover, the co-treatment of CA and cisplatin (50:5 µM) showed a rapid increase in the apoptotic cascade by increased caspase activity (1.7 folds) compared to a single administration of cisplatin. Likewise, the treatment with the same combination increases the caspase activity by >4-folds in A2780cisR cells [135]. Gherman et al. [136] reported that the caffeic acid phenethyl ester (CAPE) activates proapoptotic and epithelial-mesenchymal transition-related genes in ovarian cancer cells, A2780 and A2780cis. The CAPE is a natural derivative of CA, which has a similar structure but with an additional phenylmethyl ester group and a component of honeybee propolis [128]. Liu et al. [137] suggested that CAPE could restrain the progression of ovarian cancer via inactivating NF-κB signaling and may provide novel therapeutic regimens for ovarian cancer. They observed a remarkable decrease in the cell viability, migration, and invasion of SKOV-3 accompanied by an obstructed Ki67 and PCNA expression, nuclear translocation of p65, inhibition of IκB phosphorylation, and NF-κB p65 DNA binding activity in the CAPE treated cells [137]. In another study, Kleczka et al. [138] reported significant cytotoxicity, decreased lysosomal activity, and the total synthesis of cellular proteins in CAPE-treated OV7 serum ovarian cancer cells. Moreover, this study also reported a CAPE-induced apoptosis via dysregulation of Bax/Bcl-2 balance [138].

#### 2.2.2. *p*-Coumaric Acid

*p*-Coumaric acid (4-hydroxycinnamic acid, *p*-CA) is a phenolic acid belonging to the hydroxycinnamic acids derived from the precursor aromatic amino acids tyrosine and phenylalanine. *p*-CA is often ingested in the regular human diet from several fruits, vegetables, and cereals, such as tomatoes, carrots, onions, garlic, apples, pears, coffee, strawberries, maize, and wheat. The occurrence of *p*-CA has also been observed in conjugated forms such as amines, organic acids, alcohols, mono- or oligosaccharides, and lignin, which have stronger biological activity and is more prevalent than free form [107]. Multiple studies have shown favorable biological characteristics of *p*-CA that include low toxicity, antioxidant activities, anti-inflammatory effects, antidiabetic effects, antimicrobial activities, and anticancer effects [139,140]. The presence of a phenyl hydroxyl group has been reported as the structural feature of *p*-CA that enable free radical scavenging and antioxidant activity [141]. Various studies reported the significant role of *p*-CA in regulating the level of the antioxidant enzyme, such as superoxide dismutase, glutathione, and catalase, along with an effective conquest of lipid peroxidation and nitric oxide. p-CA has also been reported to suppress different inflammatory mediators, such as TNF-α, iNOS, and COX-2 [142]. In addition, p-CA has been reported to be effective in suppressing cellular proliferation, adhesion, and migration in various cancer models by influencing different signaling molecules, viz., NF-kB, MAPK, VEGF, and bFGF [143,144,145]. *P*-CA was evocative to be used as a prospective therapeutic drug with potential application for the treatment of OC due to the encouraging anticancer effect. An earlier study reported that a coumarin derivative, RKS262, inhibits cell cycle progression and causes proapoptotic signaling and cytotoxicity in ovarian cancer cells [146]. The proliferation of OVCAR-3 cells was adversely affected through different mechanisms, such as the downregulation of cyclin D and p21-independent inhibition of CDK-6 [146]. Interestingly, an upregulated p27 was also observed, a potential target for cancer therapeutics [147]. Akdemir et al. [148] reported the potential of *p*-CA in preventing cisplatin-induced hepatotoxicity and nephrotoxicity in Wistar adult rats. As cisplatin is a commonly used chemotherapeutic against OC, *p*-CA treatment has been suggested as a beneficial agent to attenuate oxidative stress in liver and kidney damages induced by cisplatin. Similarly, Zhang et al. [149] reported the potential effects of a coumarin compound, dicumarol, that inhibits pyruvate dehydrogenase kinase 1 (PDK1) and targets multiple malignant behaviors of ovarian cancer cells. The dicumarol empowers the PDK1 inhibition by switching the cellular glucose metabolism from aerobic glycolysis to oxidative phosphorylation, ultimately promoting different anticancer mechanisms, such as elevated ROS production, reduced MMP level, induction of apoptosis, reduced cells viability, and suppressed xenograft growth [149]. Karataş et al. [150] reported the cytotoxic effects of coumarin-substituted benzimidazolium salts against human ovarian cancer cells (A2780). They suggested it as a promising candidate for the treatment of ovarian cancers. Recently, Demir et al. [151] reported the protective effects of *p*-CA against CDDP-induced oxidative stress, inflammation, and apoptosis in the ovarian tissues of rats and suggested the usefulness of *p*-CA as adjuvant therapy in cancer management.

#### 2.2.3. Ferulic Acid

Ferulic acid (FA), (4-hydroxy-3-methoxycinnamic acid) belongs to the family of hydroxycinnamic acids found in vegetables and fruits. Its occurrence has also been reported in various medicinal plants. It rarely occurs in a free state and is typically conjugated with mono, oligosaccharides, polysaccharides, polyamines, and lipids. Multiple studies have shown a wide range of biological effects by FA against clinical conditions, including, cancer, diabetes, lung, and cardiovascular diseases [108,152,153]. It also shows hepatic, neuro, and photoprotective effects. The overall therapeutic effects of FA are generally attributed to their potential to scavenge free radicals. FA can scavenge free radicals and shield lipids and DNA from ROS by swiftly producing a resonance-stabilized phenoxy radical using the phenolic nucleus and long side chain [154]. Other therapeutic properties of FA include antimicrobial, anti-inflammatory, anticholesterolemic, vasodilatory effect, antithrombotic, radioprotective, and UV-protective effects [154].

FA had been proposed as a promising anticancer agent with potential therapeutic benefits that include antiproliferative, proapoptotic, antiangiogenic, and/or antimetastatic effects in different experimental cancer models influencing multiple molecular targets and cellular signaling pathways [108]. Karimvand et al. [155] reported a significant increase in apoptosis index characterized by decreased Bcl-2 expression and higher Bax expression after FA treatment in renal carcinoma. Activation of MMP-9 and Beclin1 has been reported to cause cell migration, invasion, metastasis, and poor prognosis in ovarian cancer [156,157]. The potential effect of FA in reducing MMP-9 mRNA expression and autophagy-related protein Beclin1 in a dose-dependent manner has also been reported [157]. In silico screening of FA with potential anticancer targets, including signal transducer and activator of transcription 3, mitogen-activated protein kinase 1, and phosphoinositide-3-kinase regulatory subunit 1 (PIK3R1), predicted potential therapeutic effectiveness [158].

#### 2.2.4. Sinapic Acid

Sinapic acid (3,5-dimethoxy-4-hydroxycinnamic acid; SA) is a derivative of hydroxycinnamic acid and is found in various vegetables and fruit species. Over the past few decades, a great deal of research has been conducted on the pharmacological properties of SA. SA is an essential antioxidant that effectively scavenges free radicals [109]. Moreover, SA has demonstrated therapeutic benefits in various clinical conditions, including diabetes, inflammation, cardiovascular disease, and cancer. It has also been reported to possess neuro, hepato, and renal protective effects against toxic agents [159,160,161].

**Figure 3:**
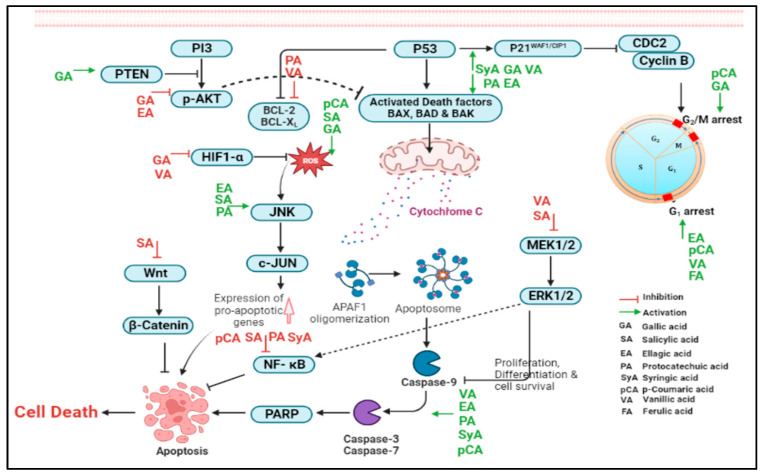
Schematic illustration of cell cycle checkpoints, survival signaling, and growth pathways and intrinsic apoptotic pathways leading to cell death mediated by phenolic acids in ovarian cancer. Activated PI3K/Akt and MEK/ERK pathways—mitogen activated kinase—promotes the survival and proliferation of ovarian tumors, while Wnt/β-catenin signaling regulates pluripotency, stemness, and differentiation of the ovarian cancer cells. ROS mediates cellular ovarian cell apoptosis by activated c-JUN/JNK signaling. The activation and inhibitory role of various phenolic acids in regulating these signaling pathways are illustrated above. (Created using Biorender basic version software).

SA has been reported to modulate cellular redox homeostasis and promotes the effective scavenging of ROS, which are commonly elevated in cancer patients, and plays an important role in all stages of chemical carcinogenesis and tumorigenesis [109,162]. Hu et al. [162] reported the anticancer effects of SA, such as potential cytotoxicity, apoptotic activity via elevation of ROS, and caspases activity (caspase-3 and caspase-9), in lung cancer cells. In one study, Balaji et al. [163] reported supplementation of SA was found to be effective in improving the oxidative burden and other abnormalities induced by 7,12-dimethylbenz[a]anthracene (DMBA). Recently, Huang et al. [164] observed inhibition in pancreatic cancer proliferation, migration, and invasion via the downregulation of AKT/Gsk-3β signal pathway by SA. AKT/Gsk-3β signal pathway has been reported as a potential therapeutic target, and its modulation could benefit OC treatment [165]. Zhao et al. [166] reported that a combination of SA and cisplatin inhibits cancer cell proliferation and migration and induces apoptosis in hepatoma cells, and suggested it as an attractive adjuvant therapy for hepatocellular carcinoma. To recapitulate our article, a schematic illustration of cell cycle checkpoints, survival signaling, growth pathways, and intrinsic apoptotic pathways leading to cell death mediated by various phenolic compounds in ovarian cancer has been provided in Figure 3.

## 3. Summary and Future Perspectives

Studies on the effectiveness of phenolic compounds (nonflavonoid subtypes) in ovarian cancer have emerged on account of their bioavailability, safety profile, and lesser side effects. The cytotoxic and anticancer findings of gallic acid indicate its high potential in ovarian cancer prevention since the therapeutic activity of GA is well tolerated by the normal ovarian cells [44]. Additionally, its mechanism of action on ovarian cancer angiogenesis has been observed via the PTEN/AKT/HIF-1α pathway [44]. Further, in vivo tumor reductions with GA corresponding to effects similar to paclitaxel treated group are interesting [47]. Thus, it is suggested as a co-adjuvant to paclitaxel in ovarian carcinoma treatment, and their combined therapy imply a novel strategy against drug resistance, demanding lower doses contrasted to paclitaxel alone [49]. The therapeutic targets of GA could probably be attributed to its potential to induce ATM/Chk2/p53 activation, COX-2/NF-kB, and GSH inhibition [47]. Taken together, anticancer studies on gallic acid highlight its medicinal importance, validate its use in chemoprevention and therapy, and brings it out as a potential candidate in ovarian cancer.

Given the proven safety record and modulating effect on signaling pathways, the possibility of using salicylic acid and its derivative for ovarian cancer treatment cannot be neglected. The multiple signaling pathways for their anticancer role includes modulation in ELK1/SRF, AP-1, MYC/MAX, NF-кB, and Wnt/β-catenin pathways [59,62]. It also interferes with several metabolic processes, including biogenesis, biogenetics, and redox regulation [61]. In line is niclosamide-targeted ovarian cancer-stem cells (CSCs), which inhibit crucial pathways such as Wnt, mTOR, and STAT3 associated with cancer metastasis and recurrence, as well as reverse platinum resistance [65]. This drug could thus be repurposed as an anticancer agent capable of inhibiting various cellular pathways, cell metabolism interruption, cellular apoptosis by inactivating MEK1/2-ERK1/2 signaling, reduced mitochondrial respiration, and aerobic glycolysis [66]. One criticism for using niclosamide as an anticancer drug is its poor water solubility and bioavailability [65]. More soluble niclosamide analogs could thus be useful for ovarian cancer combination therapy, and its nanosuspension also holds potential for OC therapeutics, but robust clinical studies are warranted [68].

Ellagic acid treatment of OC cells led to decreased cellular migration and also prevented cisplatin resistance, or cells could retain a sensitive phenotype possibly due to reduced ErbB2 and ErbB3 phosphorylation [76]. EA also inhibits angiogenesis by inhibiting growth factor VEGF secretion [43]. Ellagic acid-induced apoptotic induction (caspase-3 mediated via an increased Bax: Bcl-2 ratio and JNK and Akt phosphorylation) and cytotoxic autophagy activation (Akt inhibition and AMPK activation) enhances the efficacy of EA-based chemotherapy [74,78]. Thus, the key role in cellular apoptosis and dysregulated autophagy in ovarian cancer development and progression indicates its potential as a promising therapeutic target. The nanoformulations of EA with enhanced solubility and bioavailability could also be developed into EA-based therapeutics.

By inducing ROS reduction, p53-independent apoptosis, and autophagy, protocatechuic acid reduces the growth of ovarian cancer cells [79]. PCA also showed inhibition of cancer cell proliferation and apoptosis induction via reduced Bcl-2 expression, increased Bax level, activated JNK/p38 MAPK and Fas/FasL pathways, and had an inhibitory effect on genes associated with inflammation [84,87,88]. Oxidative stress control is one of the striking therapeutic targets in OC [89]. Hence, the antioxidant effect by PCA indicates its therapeutic efficacy against OC, and multiple clinical trials are already registered to evaluate its effectiveness in ovarian cancer [90,91]. Overall, PCA might be used as a chemopreventive agent for ovarian cancer since it affects apoptosis and autophagy.

Syringic acid is yet another phenolic found to suppress STAT3/JNK/Akt pathway along with significant growth inhibition in human ovarian teratoma cancer cells [103]. The chemoprotective action of SyA on ovarian damage is established via promoting proinflammatory cytokines TNF, IL-2, IL-6, and IL-10 [103]. It is also seen to be capable of suppressing ovarian cancer parameters, including luteinizing hormones, antimullerian hormone, estradiol, and follicle-stimulating hormone and ovarian follicles [104]. Although there is limited research on the therapeutic effects of vanillic acid on ovarian cancer, studies have shown that it interacts significantly with several targets of OC. VA inhibits multiple signaling pathways, preventing angiogenesis and suppressing cellular proliferation such as phospho-p70S6K, phospho-mTOR, phospho-4E-BP1, p-eIF4E, phospho-c-Raf, phospho- MEK1/2, and phospho-ERK1/2 [105,118]. VA has a chemopreventive efficacy against endometrial carcinoma attributed to reduced lipid peroxides and cytochrome P450 and an induced reversal of matrix metalloproteinases [114].

Caffeic acid, a phenolic acid derived from hydroxycinnamic acid, induces apoptotic cascade in ovarian cancer by increased caspase activity contrasted to cisplatin alone, enhances cisplatin cytotoxicity, and increases the amount of platinum bound to nuclear DNA [135]. Caffeic acid phenethyl ester (CAPE) could activate proapoptotic and epithelial–mesenchymal transition-related genes in ovarian cancer cells [136]. It could also restrain the OC progression via inactivating NF-κB signaling and induce apoptosis via dysregulation of the Bax/Bcl-2 balance [138]. Taken together, CA and its derivative significantly decrease the chemotherapy side effects, increase treatment effectiveness, and decrease drug resistance.

*p*-Coumaric acid (*p*-CA) also a hydroxycinnamic acid derivative could also be useful as adjuvant therapy in ovarian cancer management [151]. *p*-CA treatment is suggested as a beneficial agent to attenuate oxidative stress in liver and kidney damages induced by cisplatin-based chemotherapy [142]. The dicumarol (a coumarin compound) allows PDK1 inhibition by switching the cellular glucose metabolism from aerobic glycolysis to oxidative phosphorylation, ultimately promoting different anticancer mechanisms [149]. The potential effect of ferulic acid (FA) in OC is seen as a reduction in MMP-9 mRNA expression and the autophagy-related protein Beclin responsible for cell migration, invasion, metastasis, and poor prognosis in these cells [156,157]. In silico screening of FA with potential anticancer targets includes STAT3, MAPK1, and PIK3R1 envisaging potential therapeutic effectiveness [158]. Sinapic acid (SA), a derivative of hydroxycinnamic acid, could modulate cellular redox homeostasis and promotes the effective scavenging of ROS, commonly elevated in cancer patients, and plays an important role in all stages of chemical carcinogenesis and tumorigenesis [109,159,160,161]. Taking clues from studies on other cancer, a combination of SA and cisplatin could inhibit cancer cell proliferation and migration, induces apoptosis, and is suggested as an attractive adjuvant [166]. In addition, the AKT/Gsk-3β signal pathway is reported as a potential therapeutic target, and its modulation could benefit OC treatment [165].

The broader message from the entirety of these studies is that a phenolic acid-rich dietary intake in the form of fruits, vegetables, nuts, berries, spices, and beverages could not only prevent ovarian tumorigenesis but would also aid in halting tumor progression and metastasis. Developing drugs/compounds blocking the above elaborated molecular target proteins in ovarian cancer is worth exploring. Keeping in mind the bioavailability and bioactivity of phenolics, it would be worthy to have a deeper analysis of their antiovarian cancer effects in well-defined clinical research, taking into account the influence of various metabolic factors to obtain optimized bioavailable, well tolerated, and safe doses potentially toxic as mono- or combination therapy to develop promising in vitro profiles translating into potential leads for anticancer therapy.

Moreover, the future preclinical and clinical research on the role these phenolics in ovarian cancer should be focused toward important issues in clinical oncology: (i) precise mechanisms of action should be addressed including cellular targets and signaling pathways linked with cancer parameters (proliferation, invasiveness, metastasis); (ii) their improved bioavailability such as nanosuspension; (iii) comparative effectiveness when used alone or in combination with routine chemotherapy or as adjuvant therapy; and (iv) evaluating the anticancer potential of specific combination formulas of different phenolic compounds.

One has to understand the mechanism how these compounds accumulate in cellular organelles and tissues once they enter inside. Phenolics have the potential of modulating many biological events in ovarian cancer such as apoptosis, autophagy, angiogenesis and vascularization, cell differentiation, cell proliferation, etc. The cross talk between these phenolic compounds and the key enzymes influencing the cascade of cancer development and progression events is essential to be understood both in vitro and in vivo settings as well, to come up with new insight in ovarian cancer therapeutics and prevention. The present review thus collates results from experimental, epidemiological, and clinical studies highlighting the benefits of these phenolics as anticancer and chemopreventive agents with clinical relevance. Table 2 and Table 3 summarize the whole article highlighting the anticancer potential of various phenolic acids.

## 4. Conclusions

Due to the high costs of current chemotherapies, associated side effects, and inefficient treatment regimens, the search for alternative cancer prevention and treatment strategies has become necessary. An alternative approach to dealing with these issues is the repositioning of plant-based treatment of cancer. These treatment methods could have limited side effects, be cost-effective, and improve quality of life. Phytochemicals have two major mechanisms in which they can prevent different types of cancer. On one hand, they prevent the occurrence of three early stages of carcinogenesis, while on the other, they serve as an adjuvant to regular chemotherapy, which may prevent cancer from progressing. Thus, these compounds can be used as both preventive as well as therapeutic agents.

Phytochemicals are well established and proven reservoirs of anticancer preventive and therapeutics. It is noteworthy that a vast majority of US-FDA approved drugs in cancer are natural anticancer drugs. The salicylic acid derivative—niclosamide—is an FDA-approved drug with a good safety profile [167]. Its anticancer role is attributed as plausible inhibition of overlapping yet diverse cellular targets in different human cancers [67,168,169]. Thus, identifying and validating these cells signaling pathways that synergize or potentiate their anticancer activity will facilitate its translation as a productive and attractive agent for combination therapy. If this drug consistently gives promising results in additional preclinical studies, repurposing it would ultimately make a remarkable impact on OC patient’s lives. Future studies should be directed toward testing niclosamide or more soluble analogs as a combination regime in preclinical and clinical settings. This drug’s repurposing also has implications for chemo-resistant ovarian cancers. Thus, adding data toward this will therefore provide support in translation of investigative phytochemical drugs to clinics.

The multitargeted effects of phenolics include cell growth and proliferation, cytoskeleton, cell cycle, inflammation, angiogenesis, cell signaling, intrinsic apoptosis, and alleviating chemoresistance. Phenolic acids are potent OC preventive agents since they are well tolerated by the normal ovarian cell lineage. Results suggest that the antioxidant activity of the nonflavonoids might benefit ovarian cancer therapy.

It is acceptable that there is limited epidemiological evidence of phenolics toward ovarian cancer, but a systematic approach, including their preclinical studies with translational significance along with clinical trials employing therapeutically relevant doses, will be needed to establish their role in ovarian cancer. Research in the future should deal with formulations of phenolic acids delivery systems, their metabolic variation, and likely interactions with other agents. Nanoformulation of these compounds also holds potential as a new ovarian cancer treatment modality, but extensive clinical studies are warranted. With further advancement, synthetic or chemical approaches could be engaged to improve the activity and bioavailability of these phytochemicals. The current review outlines the several benefits of nonflavonoids, particularly phenolic acids, against EOC to improve its prognosis and/or diagnosis.

## 5. Limitation of Phenolic Acids

Epidemiological studies, however, do not always support experimental study findings. This could be due to the variable dose of phytochemicals used, their variable assimilability, and differential metabolism. It should be noted that in addition to the advantages, there may also be several drawbacks to using phytochemicals, such as their ambiguous effect on chemoprevention, the lack of information about the safety and toxicity doses, the absence of information about their potential side effects and pharmacodynamic properties. These together leads to quite often in exposure levels inadequate to address the disease process and inconsistent results regarding the molecular mechanisms of action.

## Figures and Tables

**Table 1:** Phenolic acids and their dietary plant sources.

Phenolic Compounds	Plant Sources	References
Gallic acid (GA)	Processed beverages—red wine, green tea berry/tea leaves, pomegranate root bark, gallnuts, oak bark	[34]
Salicylic acid (SA)/Salicylate derivatives	Berries—blue, boysen, logan, Nuts and dry fruits—apricot, dates, raisins Fruit—orange Beverage—black tea condiments—aniseed, cumin, hot paprika, thyme	[52]
Ellagic acid (EA)	Fruit—pomegranate, grape Berries—blackberry, raspberry strawberry, cranberry, and blueberry Nuts and dry fruits—walnuts, chestnuts, almonds	[69]
Protocatechuic acid (PCA)	Black rice, green tea, olive oil, honey, Fruits and nuts, plums, gooseberries, grapes, almonds, soybean, star anise, medical rosemary, and cinnamon As a bioactive constituent of medicinal plants, such as *Hibiscus sabdariffa*, *Ginkgo biloba*, *Hypericum perforatum*, *Cibotium barometz*, *Stenoloma chusanum*, *Ching*, and *Ilex chinensis Sims*	[80,81]
Syringic acid (SyA)	Dates, olives, pumpkin, grapes, spices, acai, red wine, palm, honey	[31]
Vanillic acid (VA)	Green tea	[105]
Caffeic acid (CA)	coffee beans, olives, berries, potatoes, carrots	[106]
*p*-Coumaric acid (p-CA)	Fruits—apples, pears, strawberries Vegetables—tomatoes, carrots, onions, garlic Cereals—maize, wheat	[107]
Ferulic acid (FA)	Fruits—pineapple, bananas Vegetables—spinach, beetroot Whole grain—oat	[108]
Sinapic acid (SA)	Fruits—oranges, grapefruits, cranberries Herbs—canola, mustard seed, rapeseed	[109]

**Table 2:** Anticancer potential of various phenolic acids (in vitro models).

Phenolic Acids	Anticancer Mechanisms	Study Model	Dose/IC_50_	References
A. Hydroxybenzoic Acids				
Gallic acid	Increased cytotoxicity and increased inhibition in cellular growth	A2780 cells	>50 µM	[42]
Gallic acid	Inhibition of VEGF, increased PTEN expression, downregulation of Akt phosphorylation and HIF-1α expression, and antiangiogenic effects	OVCAR-3 and A2780/CP70	OVCAR-3 = 66.86% A2780/CP70 = 30.10% at 40 μM	[44]
Gallic acid	ROS generation, decreased cell viability, affects cytoskeleton, cell cycle arrest, and induction of apoptosis	SKOV-3 and OVCAR-3	SKOV-3 = 50 μg/mL OVCAR-3 = 43 μg/mL	[45]
Gallic acid	Carbonic anhydrase IX protein, PI3K, and caspase-3-mediated mechanism of action, upregulation of proapoptotic proteins (Bax and Bad), p53 protein activation, and induction of apoptosis	OVCAR-3 and A2780/CP70	OVCAR-3 = 22.14 μM A2780/CP70 = 33.53 μM	[48]
Gallic acid	ROS-mediated inactivation of ERK	A2780 doxorubicin-sensitive and -resistant A2780AD	A2780 = 25% at 50 μM A2780AD = 20% at 100 μM	[49]
Salicylic acid	ELK1/SRF, AP-1, YC/MAX, and NF-кB Inhibited proliferation, migration, cell cycle progression, and induction of apoptosis	SKOV3 and HeyA8	SKOV3 = 65 HeyA8 = 53.4% at 1µM	[59]
Salicylic acid	RBPs—FXR1 and IGF2BP2, high expression of RBPs associated with reduced survival of OC patients	ES2, SKOV3, A2780, IP1, OV90, OVCAR3/4/5/8, KURAMOCHI, and OVSAHO cancer cells	0.54–3.09 μM	[60]
Salicylic acid	WNT7A/β-catenin signaling, increased E-cadherin and SLUG levels, inhibited tumor growth and progression	SKOV3.ip1 cells	0–10 μM	[63]
Salicylic acid	MEK1/2-ERK1/2; ROS-dependent JNK signaling, inhibited cell growth and induced apoptosis; reduced mitochondrial respiration as well as aerobic glycolysis	SKOV3 and HO8910	SKOV3 = 4.82 µM HO8910 = 7.12 µM	[66]
Ellagic acid	Inhibited cellular proliferation, induced G1-arrest, elevated p53 and Cip1/p21 levels, decreased cyclin D1 and E levels, induced apoptosis, increased Bax: Bcl-2 ratio, apoptotic induction, and autophagy inhibition	ES-2 and PA-1	ES-2 = 60% PA-1 = 90% at 25 μM	[74]
Ellagic acid	Inhibited tumor growth, inhibit metastasis by downregulating MMP-2 and MMP-9 expression	A2780	5, 10, and 15 μg/mL	[75]
Ellagic acid	Prevented cisplatin resistance	A2780	17.0 µM	[76]
Ellagic acid	JNK and Akt phosphorylation and induction of apoptosis	NCI/ADR RES ovarian cancer cells	Plant extract 100 and 200 µg/mL	[77]
Ellagic acid	Proliferation suppression and moderate inhibition of VEGF secretion	OVCAR-3 and A2780/CP70 cells	40 μM	[43]
Ellagic acid	Cytotoxic activity, autophagy activation mediated by Akt inhibition and AMPK activation, decreased mTORC1 and p-Akt	SKOV-3	36.6 μM	[78]
Protocatechuic acid	Antiproliferative effect	Caov-3		[92]
Protocatechuic acid	Decreased cell viability and capacity to form colonies	OVCAR3, SKOV3, and A2780 cells		[86]
Protocatechuic acid	G2/M phase cell cycle arrest, activated PARP and caspase 3, and upregulated and downregulated Bax and Bcl-2, respectively	OVCAR3		[31]
Syringic acid	Suppression of STAT3/JNK/Akt pathway, growth inhibition, modified apoptosis-related protein expression level, viz., caspase-3, 8, 9, reduced levels of proinflammatory cytokines, viz., TNF, IL-2, IL-6, and IL-10	PA-1	25 μM/mL	[103]
Vanillic acid	G1 phase arrest, inhibited proliferation, suppression of HIF-1α, and inhibition of mTOR/p70S6K/eIF4E-binding protein 1 and Raf/MEK/ERK pathways	HCT-116	30 µM	[113]
Vanillic acid	Prevent angiogenesis, suppress cellular proliferation through phospho-p70S6K, phospho-mTOR, phospho-4E-BP1, p-eIF4E, phospho-c-Raf, phospho- MEK1/2, and phospho-ERK1/2 signaling pathways	LNCaP and DU145	~25 µM	[105]
*L. sibiricus* root extract (Vanillic acid)	Enhanced caspases 3, 8, and 9 mRNA levels and reduced the mRNA levels of Bcl-2	Glioma cells	0.1–1.5 mg/mL	[115]
B. Hydroxy Cinnamic Acids				
Caffeic acid	Enhances cisplatin cytotoxicity and increases the amount of platinum bound to nuclear DNA, increases in the apoptotic cascade by increased caspase activity	A2780 and A2780cisR	5–20 µM	[135]
Caffeic acid	Activates proapoptotic and epithelial–mesenchymal transition-related genes in ovarian cancer	A2780 and A2780cis	A2780 = 34.98 µM A2780cis = 58.01 µM	[136]
	Restrains the progression of ovarian cancer via inactivation of NF-κB signaling, decreased cell viability, migration, and invasion accompanied by an obstructed Ki67 and PCNA expression, nuclear translocation of p65, inhibition of IκB phosphorylation, and NF-κB p65 DNA binding	SKOV-3	50 μM	[137]
Caffeic acid	Increased cytotoxicity, decreased lysosomal activity, and the total synthesis of cellular proteins, induced apoptosis via dysregulation of Bax/Bcl-2 ratio	OV7 serum ovarian cancer cells	XTT-142.58 µM NR-81.43 µM SRB-80.08 µM	[138]
p-Coumaric acid	Inhibits cell cycle progression, induces proapoptotic signaling, increased cytotoxicity, downregulation of cyclin D and p21-independent inhibition of CDK-6	OVCAR-3	75% cytotoxicity with 5 µM	[146]
p-Coumaric acid	Increased cytotoxicity	A2780	~10 µM	[150]
Ferulic acid	Increased apoptosis index, decreased Bcl-2 expression and higher Bax expression	ACHN	39.5 μM	[155]
Ferulic acid	Reduced MMP-9 mRNA expression and autophagy-related protein Beclin1 levels	Hela and Caski	Hela = 88.3% Caski = 85.4% with 2.0 mM	[157]
Sinapic acid	Increased cytotoxicity, apoptotic activity via elevation of ROS and caspases activity (caspase-3 and caspase-9)	A549	50 µM	[162]
Sinapic acid	Downregulation of Akt/Gsk-3β signal pathway	Pancreatic cancer cells		[164]
Sinapic acid	Inhibits cancer cell proliferation and migration and induces apoptosis	HepG2	1795 μM	[166]

**Table 3:** Anticancer potential of various phenolic acids (animal models and humans).

Phenolic Acids	Anticancer Mechanisms	Study Model	Dose/IC_50_	References
A. Hydroxybenzoic Acids				
Gallic acid	ATM/Chk2/p53 activation, COX-2/NF-kB, GSH inhibition and inhibition of tumor lesions development	Mice	50 mg/kg of body weight	[45]
Gallic acid	Reduced MDA production and oxidative stress (SOD, CAT, GSH-Px, TAOC activity) and increased serum antioxidant enzymes activity	Rats	Plant extract 150 mg/kg body weigh	[50]
Salicylic acid	Wnt/β-catenin, increased cytotoxicity in combination with carboplatin	34 patients’ ascites with primary ovarian cancer		[62]
Salicylic acid	Wnt, mTOR, and STAT3, antiproliferative, cell cycle arrest, induced apoptosis, and platinum resistance reversal	Tumor spheres from ascites of all OC patients who were suspected to have ovarian cancer and scheduled to undergo surgery and cells from a chemo-resistant, patient-derived xenograft	0.1 to 5 µM	[64]
Salicylic acid	Decreased expression of p-MSK1, p-MEK1/2, and p-ERK1/2, and suppressed tumor growth	Xenograft tumor model	20 mg/kg body weight	[66]
Ellagic acid	Inhibited tumor growth, inhibited metastasis by downregulating MMP-2 and MMP-9 expression	Nude mice	50 mg/kg body weight	[75]
Salicylic acid	Decreased expression of p-MSK1, p-MEK1/2, and p-ERK1/2, and suppressed tumor growth	Xenograft tumor model		[66]
Salicylic acid	WNT7A/β-catenin signaling, increased E-cadherin and SLUG levels, inhibited tumor growth and progression	SKOV3.ip1 cells and xenograft mouse model	200 mg/kg body weight	[63]
Protocatechuic acid	Induced apoptosis, maintained cell proliferation and mitochondrial function, reduced ROS production, and increased GSH expression through PTEN and FOXO3a proteins	Mice	20 and 50 mg/kg body weight	[93]
Syringic acid	Chemoprotective action, decreased nitric oxide, myeloperoxidase, catalase, glutathione, glutathione peroxidase, superoxide dismutase, and malondialdehyde levels in both serum and ovarian tissue. Suppressed luteinizing hormones, antimullerian hormone, estradiol, follicle-stimulating hormone, and ovarian follicles. downregulated cytokines, inflammatory mediators, and caspase-3	Swiss albino Wistarrats	5–20 mg/kg body weight	[104]
B. Hydroxy Cinnamic Acids				
p-Coumaric acid	Prevent cisplatin-induced hepatotoxicity and nephrotoxicity	Wistar rats	100 mg/kg body weight	[148]
p-Coumaric acid	Inhibit oxidative stress, inflammation, and apoptosis	Rat	4 mg/kg	[151]
Sinapic acid	Improve oxidative burden and other abnormalities	Rats	80 mg/kg body weight	[163]

Akt—serine/threonine kinase family; AMPK—AMP-activated protein kinase; AP—activator protein; ATM—ataxia telangiectasia mutated; Bad—BCL2-associated agonist of cell death; Bax—Bcl-2-associated X protein; Bcl—B-cell lymphoma; BP—binding protein; CDK—cyclin-dependent kinases; Chk—checkpoint kinases; COX—cyclooxygenase; DNA—deoxyribonucleic acid; ELK—ETS transcription factor; ERK—extracellular signal-regulated kinase; FOXO3—forkhead box protein O3; FXR1—Farnesoid X receptor; GSH—glutathione; Gsk—glycogen synthase kinase; HIF—hypoxia; IF—inducible factor; IGF—insulin-like growth factor; IL—interleukin; IκB—inhibitor of nuclear factor kappa B; JNK—c-Jun N-terminal kinases; Ki67—marker of proliferation; MEK—mitogen-activated protein kinase; MMP—matrix metalloproteinases; mTOR—mammalian target of rapamycin; MSK—mitogen- and stress-activated protein kinase; PCNA—proliferating cell nuclear antigen; PI3K—phosphoinositide 3-kinase; PTEN—phosphatase and tensin homolog; Raf—rapidly accelerated fibrosarcoma; RBPs—RNA-binding proteins; RNA—ribonucleic acid; ROS—reactive oxygen species; SOD—superoxide dismutase; SRF—serum response factor; STAT3—signal transducers and activators of transcription; TNF—tumor necrosis factor; VEGF—vascular endothelial growth factor.

## Data Availability

Data sharing not applicable.
